# Prevalence of risk factors for acquiring measles during the 2011 outbreak in Quebec and impact of the province-wide school-based vaccination campaign on population immunity

**DOI:** 10.1371/journal.pone.0186070

**Published:** 2017-10-11

**Authors:** Marie-Noëlle Billard, Gaston De Serres, Marie-Claude Gariépy, Nicole Boulianne, Eveline Toth, Monique Landry, Danuta M. Skowronski

**Affiliations:** 1 Centre de recherche du CHU de Québec—Université Laval, Quebec City, QC, Canada; 2 Institut national de santé publique du Québec, Quebec City, QC, Canada; 3 Ministère de la santé et des services sociaux du Québec, Montreal, QC, Canada; 4 British Columbia Center for Disease Control, Vancouver, BC, Canada; Universidade Nova de Lisboa Instituto de Higiene e Medicina Tropical, PORTUGAL

## Abstract

**Background:**

A large measles outbreak occurred in Quebec, Canada, in 2011. Although nearly two-thirds of the cases occurred in only two health districts, a mass vaccination campaign targeting all Quebec elementary and high school students without valid two-dose history was undertaken to prevent future outbreaks. We compared rates of non-vaccination and age at first measles vaccine dose among students in the two most-affected districts and the rest of the province and estimated the improvement in overall student measles immunity due to the mass school-based vaccination campaign.

**Methods:**

Data were extracted from the provincial vaccination registry for students in kindergarten to grade 11 during the 2011/2012 school year. A telephone survey was conducted in three sub-groups: students whose first measles vaccine dose recorded in the vaccination registry was received during the 2011 school vaccination campaign; students with no dose recorded in the registry whose parents refused receipt during the school campaign; and students with no dose recorded in the registry and no information about parental consent/refusal during the school campaign.

**Results:**

Neither the prevalence of being non-vaccinated nor a younger age at first pediatric dose were higher in the two most-affected districts versus the rest of the province. The school campaign vaccinated nearly 8% of all students including 7% who previously received at least one dose. Before the outbreak, 3% of students were not vaccinated and one-third of these (1%/3%) were vaccinated during the campaign. The campaign likely increased the absolute school population immunity by just 1.7%.

**Conclusion:**

The concentration of measles cases in the two most-affected health districts during the large Quebec outbreak is not explained by more students who were unvaccinated or who had received their first vaccine dose at a younger age. The vaccination campaign reached one-third of unvaccinated students and only marginally improved population immunity.

## Introduction

In spring 2011, Quebec, Canada, was affected by the largest measles outbreak in North America in over a decade that included 678 confirmed cases [1]. The outbreak started in a high school when a staff member became ill after returning from holiday outside Canada. Half of the high school cases were unvaccinated and the other half had received two doses of measles vaccine. In outbreak investigation, the two main risk factors that were identified for acquiring measles were being unvaccinated (4.7% of students were unvaccinated, RR≈20) and, for twice immunized students, having received the first dose at an early age (RR≈4 for 12 vs ≥15 months of age)[1,2]. The province is divided in 18 regions subdivided into 166 health districts. Despite the spread of measles to the community and into 8 of the 18 health regions, Montreal (the largest city of the province) was not affected and the outbreak remained concentrated in two of the 12 health districts of the most-affected region. These two districts accounted for 87% of all cases in that region and 65% of all provincial cases. At the time of the outbreak there was no provincial vaccination registry and it was not possible to assess whether the prevalence of these risk factors was higher in these districts compared to other regions and Montreal.

To prevent further outbreaks, a province-wide school-based vaccination campaign against measles was conducted between November 15, 2011 and June 30, 2012 in all elementary and high schools[3]. A provincial electronic vaccination registry that included all Quebec residents born since 1970 was created. The registry included all measles vaccination data from existing regional and local vaccination registries, data from vaccination booklets and doses administered. It also included data about parental refusal to vaccination during the campaign if their child was otherwise eligible, a variable not meant to reflect a permanent vaccine refusal. Students with no written proof of two valid doses of measles vaccine were eligible for vaccination in this mass school-based campaign. To be considered valid the first dose must have been administered at ≥12 months of age and the second dose given ≥ 28 days after the previous dose. Before the mass campaign, 14.9% of students had no dose recorded in the registry. Most of these students had lost or did not have their vaccination booklet but were expected to have been vaccinated based on vaccination coverage surveys in toddlers conducted every other year since 2006 showing that 97% had received their 1^st^ dose by 2 years of age and 90% had their 2^nd^ dose [4–8].

The first objective of this study was to use the registry data to compare vaccination coverage and age at first dose between the two most-affected districts during the 2011 outbreak and the rest of the region or the rest of Quebec. The second objective was to estimate the proportion of unvaccinated individuals immunized during the campaign, the absolute increase in population immunity brought about by the campaign and the proportion still unvaccinated after the campaign assessed through a telephone survey of students with no dose recorded prior to November 2011.

## Methods

This study was legally mandated under the Quebec Public Health Act by the Chief Medical Officer as part of the 2011 measles outbreak investigation and was not subject to ethics board review.

To estimate vaccine coverage and age at first dose by location, on September 5, 2013 we extracted data from the registry on individuals born between October 1, 1994 and September 30, 2006 representing the birth cohorts of students in kindergarten to grade 11 during the 2011/2012 school year. To estimate the remaining proportion of unvaccinated individuals we conducted a telephone survey between February 17, and 26, 2014 in three sub-groups of the entire school population of the province.

1. Students randomly selected among those who received their first dose recorded in the vaccination registry during the school vaccination campaign (1^st^ dose group)
2. Students randomly selected among those with no dose recorded in the registry in September 2013 whose parents refused the vaccination offer during the school campaign.(Refusal group)
3. Students randomly selected among those with no dose recorded in the registry in September 2013 and no information about parental response during the school campaign.(No-Info group)

Trained interviewers of a polling firm called parents of these children for a 4-item questionnaire with the objective of recruiting at least 200 participants per group. After obtaining verbal consent, parents were asked if their child had been vaccinated against measles prior to the school campaign (for the 1^st^ dose group) or since birth (for the refusal and the no-info groups). If the child had not been vaccinated, parents were asked if this was due to parental choice, medical contraindication, or another reason. Parents were asked if their child had received other childhood vaccines and was born within or outside Canada. Parents who did not want to participate in the full questionnaire were only asked if their child had ever been vaccinated.

### Statistical analysis

Analyses were stratified by age groups 5-11years and 12-17years, broadly representing elementary and high school ages. Age at vaccination in months was derived by subtracting the birth date from the vaccination date, divided by 30.44 and rounded to the lower whole number except for children vaccinated exactly on their first birthday (365/30.44 = 11.99 mo.) whose age at vaccination was defined as 12 months. Measles vaccine coverage before the 2011 outbreak was estimated counting only doses administered before May 1, 2011. To estimate the absolute proportion of unvaccinated children in the school population, we multiplied the proportion of unvaccinated students reported by their parents in each of the three subgroups of the telephone survey by the weight (%) of the corresponding subgroup in the school population (number of students in the subgroup/total number of students in the province). The confidence intervals were calculated using the SURVEYFREQ Procedure in SAS 9.3.

To estimate the absolute increase in immunity in the school population brought by the campaign, we used results from the investigation of the first high school affected by the 2011 measles outbreak during which the attack rate was 82% (50/61) in unvaccinated students, 3.4% (3/89) in students with written proof of only one dose, 7.1% (2/28) in those reported as vaccinated but with no written proof and 16.7% (2/12) in those with an unknown vaccination status[2]. The vaccine effectiveness (VE) calculated as (1-RR vacc/unvaccinated) was 95.9%, 91.3% and 79.6% respectively. For each vaccination status, we then assumed that the proportion susceptible (PS) was equal to (100%—VE) or 4.1%, 8.7%, 20.4% respectively and 100% for unvaccinated students. We optimistically assumed that all susceptible students receiving a single dose during the campaign became fully protected and calculated the absolute increase in immunity as:

Absolute immunity increase = (%one dose x 4.1%) + (%vaccinated no written proof x 8.7%) + (%unknown status x 20.4%) + (%unvaccinated X 100%)

## Results

The vaccination registry included 1,001,848 individuals born between October 1, 1994 and September 30, 2006. There were 5,062 (0.5%) exclusions: 2,347 had data entry errors (182 with >5 doses registered and 2,165 with a vaccination date inconsistent with their birth date or dates of previous doses) and 2,715 were residents from Northern Quebec where data were not entered in the registry. The analysis included the 996,786 remaining students.

On May 1, 2011, at the onset of the outbreak, 78.6% of students in the province had received two doses of measles vaccine (76.7% two valid doses), 6.5% had received only one dose (6.3% one valid dose) and 14.9% (n = 148,431) had no measles vaccine information. (Table 1) Overall and in the analysis stratified for elementary (6-11years) and high school (12-17years) age categories, vaccine coverage with two and one doses were slightly but significantly lower in the two most-affected health districts than in the other districts of their region but higher than in the rest of the province.(Table 2). More high school than elementary students had no information (18.6% vs 12%). The median age at first dose was significantly older in the two most-affected health districts (12.8 months) than in the other districts (12.2 months, Wilcoxon/Mann-Whitney p<0.001) of the region or in the rest of the province (12.5 months, p<0.001). In Montreal, the largest city of the province, there were more students with no vaccine data, the vaccine coverage with one and two doses was lower and children received their first dose at a younger age than in the two most-affected health districts (Table 2).

**Table 1:** Measles vaccine coverage before the outbreak in May 2011 and after the mass vaccination campaign in September 2013 and number of students vaccinated during the outbreak and during and after the vaccination campaign.

			Number of students administered at least one dose of measles vaccine between May 1, 2011 and September 4, 2013			
Number of recorded doses	Number of valid doses	Vaccination status before the outbreak, May 1, 2011	During the outbreak, from May 2, to November 14, 2011	During the mass vaccination campaign, from November 15, 2011 to June 30, 2012	After the mass vaccination campaign, from July, 1, 2012 to September 5, 2013	Vaccination status on September 5, 2013
		N = 996,786	N = 7,614	N = 79,949	N = 4,056	N = 996,786
**≥ 2**	**≥ 2**	76.7 %	667	6 103	291	83.7 %
	**1**	1.9 %	414	8 389	361	1.1 %
	**0**	0.02 %	11	54	4	0.01 %
**1**	**1**	6.3 %	3 783	28 299	997	4.1 %
	**0**	0.2 %	229	1 245	115	0.1 %
**0**	**NA**	14.9 %	2 510	35 859	2 288	11.0 %

**Table 2:** Age at first dose of measles vaccine and vaccine coverage among elementary students, high school students and the whole student population according to the geographic area of residence in May 2011, before the outbreak.

	Most-affected region		Rest of the Province	
	Two most-affected health districts	Other health districts	All regions	Montreal
**Total (N)**	21870	35995	938921	211072
2 doses (%)	17819 (81.5%)	31475 (87.4%)	734188 (78.2%)	144095 (68.3%)
1 dose (%)	1820 (8.3%)	1720 (4.8%)	61333 (6.5%)	16701 (7.9%)
No dose recorded (%)	2231 (10.2%)	2800 (7.8%)	143400 (15.3%)	50276 (23.8%)
*Parental refusal*	550 (2.5%)	1020 (2.8%)	21232 (2.3%)	4853 (2.3%)
*Other*	1681 (7.7%)	1780 (4.9%)	122168 (13.0%)	45423 (21.5%)
**Elementary schools (N)**	12441	19591	531111	123040
2 doses (%)	10329 (83.0%)	17183 (87.7%)	432925 (81.5%)	90182 (73.3%)
1 dose (%)	970 (7.8%)	914 (4.7%)	33088 (6.2%)	9757 (7.9%)
No dose recorded (%)	1142 (9.2%)	1494 (7.6%)	65098 (12.3%)	23101 (18.8%)
**High schools (N)**	9429	16404	407810	88032
2 doses (%)	7490 (79.4%)	14292 (87.1%)	301263 (73.9%)	53913 (61.1%)
1 dose (%)	850 (9.0%)	806 (4.9%)	28245 (6.9%)	6944 (7.9%)
No dose recorded (%)	1089 (11.5%)	1306 (8.0%)	78302 (19.2%)	21175 (24.1%)
**Age at first dose** *				
<12 months	231 (1.2%)	520 (1.6%)	31009 (3.9%)	11299 (7%)
12 months	10520 (53.6%)	22636 (68.2%)	478444 (60.1%)	83740 (52.1%)
13 months	3153 (16.1%)	4641 (14.0%)	115781 (14.6%)	21217 (13.2%)
14 months	1443 (7.3%)	1696 (5.1%)	44982 (5.7%)	9356 (7.2%)
≥15 months	4292 (21.9%)	3702 (11.2%)	125305 (15.8%)	35184 (21.9%)
Median (Q1-Q3)	12.8 (12.2–14.5)	12.2 (12.4–13.3)	12.5 (12.2–13.6)	12.6 (12.2–14.4)

* % among vaccinated students

During the outbreak period (between May 1 and November 14, 2011), 7,614 students were vaccinated (8,324 doses) against measles. (Table 1) During the school vaccination campaign (November 15, 2011 to June 30, 2012) the registry indicated that 79,949 (8.0% of all students) students were vaccinated (106,188 doses) including 34,626 (43%) who received their first recorded dose during the campaign (1^st^ dose group). On September 2013, 109,665 students (11%) still had no dose recorded in the registry: 21,785 (2.2% of all students) had a parental refusal to the vaccination offer (Refusal group) and 87,880 (8.8% of all students) had no information indicating parental refusal (No-info group). The proportion of students with parental refusal was lower in the two most-affected health districts than in the other districts of their region (2.4% vs 2.8%, p = 0.02) but higher than in the rest of the province (2.2% p = 0.005) whereas the reverse trend was observed for the No-info group (4.1% vs 2.0% vs 9.2% respectively).

**Fig 1:**
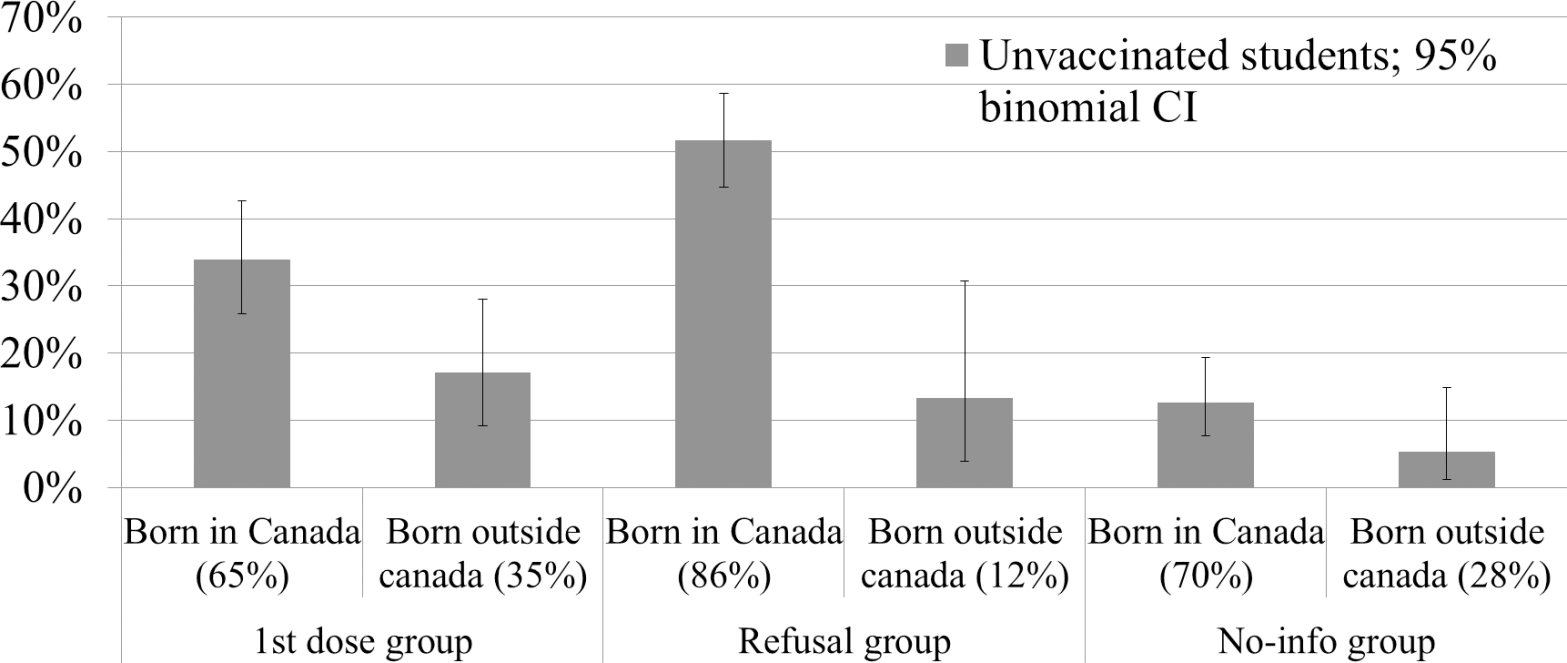
Proportion of unvaccinated students in each group sample by country of birth (Canada /outside Canada).

For the telephone survey, among eligible individuals who were reached, the participation rate was 93% (200 participants) for the 1^st^ dose group, 94% (243 participants) for the Refusal group and 95% (202 participants) for the No-info group.(Table 3) Overall, 28% (CI95% 22% to 34%) of the students in the 1^st^ dose group were reported by their parents as being unvaccinated in November 2011 before the campaign whereas 47% (CI95% 41% to 54%) and 11% (CI95% 7% to 15%) were still unvaccinated in February 2014 at the time of the survey in the Refusal and No-info groups respectively. In the Refusal group, 45% were reported as already vaccinated against measles. When extrapolated to the school population, each group had ~10 000 unvaccinated students representing 1% of all students. (Table 3) Most students belonging to the 1^st^ dose (87%) and No-info groups (90%) had received other childhood vaccines according to their parents compared to only 59% in the Refusal group. In the Refusal group, 17% of students reported as unvaccinated against measles had received other childhood vaccines compared to 94% for those not unvaccinated against measles.(Table 3) A majority of parents who reported their child as unvaccinated against measles stated that it was their choice (73.2%, 95.7% and 86.4% in the 1^st^ dose, Refusal and No-info groups respectively). The percentage of participants reported as unvaccinated among those living in Montreal was similar in the 1^st^ dose group (25%, 18/72), but much lower in the Refusal group (20%, 9/46) and in the No-info group (4%, 3/82) compared to the entire groups. The percentage reported by parents as unvaccinated was lower among students born outside Canada than among those born in Canada: 17% vs 34% in the 1st dose group, 13% vs 52% in the Refusal group and 5% vs13% in the No-info group but the difference was statistically significant only for the No-Info group.(Fig 1)

**Table 3:** Participation in the telephone survey and proportion of unvaccinated students per subgroup and in the student population.

		No recorded dose	
	1^st^ recorded dose given during the campaign	Parental refusal to vaccination during the campaign	No information about parental response
	(1st dose group)	(Refusal group)	(No-Info group)
**Total number in registry (% of all students)**	34,626 (3.5%)	21,785(2.2%)	87,880 (8.8%)
Randomly selected (N)	363	378	375
Invalid phone number	91	56	84
No response	58	64	79
Refusal	14	15	10
**Participants with questionnaire completed**			
N(% participation)	200 (93%)	243 (94%)	202 (95%)
**Measles vaccination status**			
Unvaccinated (95% Binomial CI)	28% (21.8%–34.2%)	47.3% (41.1–53.6)	10.9% (6.6%–15.2%)
*% unvaccinated by parental choice*	*73*.*2%*	*95*.*7%*	*86*.*4%*
Already vaccinated	48.5%	45.3%	81.2%
Unknown	23.5%	7.4%	7.9%
**Received other childhood vaccines**			
Overall	87%	59.2%	89.5%
*Unvaccinated*	62.5%	16.8%	22.7%
*Others*	96.5%	96.1%	96.1%
**Unvaccinated among all students**			
Number (% unvaccinated x total number in registry)	9695(28% x 34626)	10304 (2.2% x 21785)	9579 (8.8% x 87880)
% (% unvaccinated x % of all students) 95% CI	1% (28% x 3.5%) 0.8%-1.2%	1% (2.2% x 47.3%) 0.9%-1.2%	1% (10.9% X 8.8%) 0.6%-1.3%

As 3.5% of the school population belonged to the 1^st^ dose group, if 28% were actually completely unvaccinated as reported by parents during the phone survey, then the campaign achieved a 1% (28% x 3.5% = 1%, CI95% 0.9% to 1.2%) absolute reduction of unvaccinated students in the school population. Although 11% of students had no dose recorded in September 2013, only 2% were unvaccinated according to parental report including 1% (2.2% school population x 47% unvaccinated) in the Refusal group and 1% (8.8% school population x 11% unvaccinated) in the No Info group.

A written proof of two valid measles vaccine doses was available for 77% of all students. The vaccination campaign targeted the remaining 23%: at least one dose was administered to 8% (35% of the targeted students) but the absolute increase in school-population immunity was much lower. The vaccination of 45,323 students (57% of students vaccinated during the campaign, 4.5% of the school population) who were already immunized with at least one dose before the campaign according to the registry added 0.2% (4.5% school population X 4.1% susceptibility x 100% immunity with additional dose) to the population immunity. Based on the phone survey results, the other 34,626 students (3.5% of the school population) vaccinated during the campaign that had no previous dose recorded could be divided as: 1% unvaccinated, 1.7% vaccinated with no written proof and 0.8% with an unknown status (Table 4). Their vaccination during the campaign increased the absolute population immunity by 1%, 0.1% and 0.2% respectively. Accordingly, the mass school-based campaign increased the absolute measles immunity in the school population by just 1.5% in total.

**Table 4:** Absolute increase in the school population immunity following the mass campaign by vaccination status before the campaign.

Vaccination status before the campaign as recorded in the registry	% immune before mass vaccination campaign*	Absolute Increase in immunity with additional vaccination**	% of the school population	Absolute Increase in immunity in the school population (%)
**≥1 dose**	95.9%	4.1%	4.5% (45323/996786)	4.1% x 4.5% = 0.2%
**No dose**			3.5% (34626/996786)	
Unvaccinated *(28%†)*	0%	100%	1.0% (3.5% x 28%†)	100% x 1.0% = 1.0%
Vaccinated no proof *(48*.*5%†)*	84.9%	8.7%	1.7% (3.5% x48.5%†)	8.7% x 1.7% = 0.1%
Unknown status *(23*.*5%†)*	73%	20.4%	0.8% (3.5% x 23.5%†)	20.4% x 0.8% = 0.2%
**Total**			8.0% (79949/996786)	0.2% +1.0%+0.1%+0.2% = 1.5%

* Estimated from vaccine effectiveness in the first affected high school

** Assuming 100% immunity after additional vaccination

† According to the phone survey (1^st^ dose group)

## Discussion

While the clustering of 65% of measles cases in two health districts during the 2011 outbreak suggested a higher proportion of susceptible students in this area than in the surrounding regions, the prevalence of the two main risk factors associated with measles (being unvaccinated and a younger age at 1st dose) did not differ much between these two districts and the rest of the province. To prevent future outbreaks, a broad intervention was undertaken in all schools of the province and vaccinated nearly 8% of the students. According to the phone survey, 3% of students were unvaccinated before the campaign; the 1% who agreed to be vaccinated contributed to two-thirds of the absolute increase in the school population immunity. Among the 2.2% of students with a parental refusal to vaccination during the campaign, 47% were already vaccinated against measles and most (>90%) had also received other childhood vaccines. Despite intense efforts to collect vaccination status, 11% of students still had no data after the campaign, including 2% who remained unvaccinated according to the parental phone survey. This study illustrates the difficulty of immunizing unvaccinated individuals, the small improvement in population immunity obtained even with a mass campaign targeting a discrete and accessible school-based cohort, the importance of keeping track of vaccination data and the challenges to maintain elimination.

Measles elimination requires a high level of protection to maintain population immunity below the epidemic threshold[9]. The size and duration of the 2011 outbreak suggest that the school population in the most-affected region was not far below this threshold. There was little difference in the proportion of unvaccinated students and age at first dose of measles vaccine between the two most-affected districts and the rest of the province, suggesting that immunity in the school population of the entire province was also close to the epidemic threshold. When population immunity is close to the epidemic threshold, even small changes in population immunity can have an important impact on the risk of large-scale outbreaks. The large campaign certainly improved the safety margin but the estimated 1.5% increase of the school population immunity was based on optimistic assumptions, which shows the limit of even broad-scale interventions.

Unvaccinated individuals are the prime target of any intervention to prevent measles transmission. While lack of vaccination may sometimes be due to oversight or a missed visit, in general it results from an active decision made by parents, as shown in this study by the high proportion of survey participants reporting parental choice as the reason for non-vaccination. The unvaccinated students that accepted to be immunized during the campaign appear to come from families less opposed to vaccination in general. These students had more often received their other childhood vaccines than those whose parents refused or did not reply to the vaccination offer during the campaign (63%, 16% and 23% respectively). Parents who did not know their child vaccination status against measles reported that 85% to 96% of them had received their childhood vaccines, suggesting that they were not opposed to vaccination globally or vaccination against measles particularly. For the campaign to have reduced from 3% to 2% the proportion of unvaccinated students was a success but those still unvaccinated will likely never get the vaccine voluntarily. This is unfortunate as two-third (1%/1.5% = 67%) of the absolute gain in population immunity was obtained by immunizing unvaccinated students whereas vaccination of the other 7% of students for whom parents reported a single prior dose, or for whom there was no written proof or unknown status (i.e. 4.5% + 1.7% + 0.8%, respectively) stood to improve population immunity by only 0.7% (i.e. 0.2% + 0.1% + 0.2%, respectively).

The absence of spread of the 2011 outbreak to Montreal (located less than 100 km from the two most-affected districts) more likely resulted from chance or a lack of contacts with cases of the affected regions than superior immunity. Despite a much larger, more dense and diverse population than the two affected districts [10], Montreal had higher prevalence of students with no dose recorded or with a young age at first dose. The latter can be partly explained by the proportion of immigrants coming from world regions where MMR is recommended before 12 months. According to the 2011 Canadian census, 9% of Montreal residents under 15years-old were immigrants; including 2.7% and 2.3% born in Africa and Asia respectively[10]. Students born outside Canada were overrepresented in the three groups of the phone survey. This may result from either a higher proportion unvaccinated or a greater proportion that had lost their vaccination records. While we cannot rule out the former hypothesis, the latter seems more probable as in the three groups of the phone survey there was a lower proportion of students reported by their parents as unimmunized when born outside Canada compared to those born in Canada. This result relies on parental information and we cannot rule out that immigrant parents may be more fearful than Canadian parents to reveal lack of vaccination of their children.

The median age at first dose decreased gradually from 12.8 to 12.4 months of age in children born between 1994 and 2006. This probably comes from the efforts spent over that period to improve vaccine schedule timeliness. There was a greater proportion of students with no vaccination information in high schools than primary schools. Although we cannot rule out a greater proportion of unvaccinated among older students, this is more likely the result of the longer time lapse since measles vaccination with resulting greater chances of losing their vaccination record. Even with substantial investment to collect the vaccination status of every student, when the information is lost, not much can be done but to offer revaccination with two doses. For parents who knew their child had received all childhood vaccines but could not provide written proof, this offer was not well accepted. They likely perceived this as an administrative process rather than an intervention to protect their child. The implementation of an electronic vaccination registry could address this problem, but such registry requires great efforts to enter and validate all data and maintain high quality of information and completeness.

This study presents some methodological limitations. Analyses used the school cohort for the 2011/2012 school year. As grade level was not available for this year, we identified the cohort using birth dates and age criteria of inscription in school in Quebec (at least 5 years old on October 1, 2011 and until 17 years old) as a proxy. Some younger children could already be in school but were not included whereas some teenagers 16- 17years-old could have left school but were included. However, these groups represent a small proportion of the overall sample and are unlikely to have substantially influenced results. As the immunization registry only had data on measles vaccine, it was not possible to evaluate if students with no vaccine dose recorded had received other routine childhood vaccines. The main study result about the percentage of unvaccinated students is based on the assumption that this was generally the result of an active decision made by parents who would therefore be well aware of the unvaccinated status of their child and would not hide it from the interviewer. It is possible that social desirability bias may have led to an under-estimation of the proportion of unvaccinated students. However, the participation rates in the telephone survey were high with a small percentage of refusals in each of the three groups and parents of unvaccinated were quite adamant that it was indeed their decision. As the survey was conducted two years after the vaccination campaign, it is possible that recall bias may have reduced the proportion of students reported as unvaccinated in the survey and underestimated the absolute increase in population immunity. To estimate the increase in population immunity we assumed that the proportion of protected students in the entire province was similar to the vaccine effectiveness found in the outbreak investigation of the first affected high school located in one of the two most-affected health districts. Our findings are similar to vaccination coverage surveys conducted every other year in Quebec since 2006 showing that 2%-3% of children were unvaccinated against measles at 24 months of age although those surveys are also susceptible to social desirability bias[4–8].

In conclusion, students in the two most-affected health districts did not have substantially different vaccination coverage or age at first dose than the other health districts in the region of the rest of the province. Other factors must have therefore contributed to the concentration of cases in these districts including favorable circumstances for transmission at the moment of the index case’s importation rather than greater vulnerability compared to surrounding regions. The school-based vaccination campaign reduced the proportion of unvaccinated students by about one third. Although 11% of students did not have any record of a measles vaccine dose in the electronic registry one year after the end of the campaign, only 2% appear to have remained unvaccinated according to parental report. This study illustrates the difficulty of immunizing unvaccinated individuals, the small improvement in population immunity to be achieved by a mass campaign, the importance of keeping reliable track of vaccination data to improve the efficiency of outbreak response and minimize wasted investment, and the challenges to be faced in maintaining measles elimination.

## Supporting information

S1 Dataset Parental survey dataset–February 2014. (XLSX)
